# Practice Patterns in Pediatric Cardiothoracic Presurgical Conferences: A Multicenter Survey Study

**DOI:** 10.1007/s00246-025-04008-y

**Published:** 2025-08-29

**Authors:** Britney Reed, Poonam Puranik, Andrew Rodenbarger, Mira Trivedi, Dilachew A. Adebo, Gary Beasley, Louis Bezold, M. Jay Campbell, Michael R. Carr, Joshua Daily, Mark DeBrunner, Erica Del Grippo, Carlen G. Fifer, Timothy M. Hoffman, Susan R. Hupp, Joshua D. Kurtz, Gira Morchi, Carl Owada, Renuka Peterson, Michael D. Puchalski, Ryan A. Romans, Arwa Saidi, Rajesh U. Shenoy, Anoop K. Singh, Robert D. Tunks, Thomas M. Yohannan, Carolyn M. Wilhelm, Jyoti K. Patel

**Affiliations:** 1 IU School of Medicine, Indianapolis IN, Indianapolis, USA; 2 Pediatric Cardiology, Riley Hospital for Children, Indianapolis IN, Indianapolis, USA; 3 Children’s Heart Institute, University of Texas Health Science Center at Houston, Houston, TX USA; 4 Department of Pediatrics, Division of Pediatric Cardiology, University of Iowa Carver College of Medicine, Iowa City, IA USA; 5 Department of Pediatrics, Joint Pediatric Heart Program, University of Kentucky College of Medicine, Lexington, KY USA; 6 Duke University School of Medicine, Durham, NC USA; 7 Ann & Robert H.Lurie Children’s Hospital Chicago, Chicago, IL USA; 8 Arkansas Children’s Hospital, Little Rock, AR USA; 9 Pediatric Cardiology, Children’s Hospital of Pittsburgh of UPMC, Pittsburgh, PA USA; 10 Nemours Cardiac Center, Wilmington, DE USA; 11 Michigan Congenital Heart Center, University of Michigan, Ann Arbor, MI USA; 12 University of North Carolina, Chapel Hill, NC USA; 13 Duke Children’s Hospital, Duke University, Durham, NC USA; 14 Division of Pediatric Cardiology, University of Louisville, Louisville, KY USA; 15 Pediatric Cardiology, Children’s Hospital of Orange County, Orange, CA USA; 16 Department of Cardiology and Cardiothoracic Surgery, Valley Children’s Hospital, Madera, CA USA; 17 Department of Pediatrics, Saint Louis University School of Medicine, Saint Louis, MO USA; 18 Johns Hopkins All Children’s Hospital, St. Petersburg, FL USA; 19 Ward Family Heart Center, Children’s Mercy Kansas City, Kansas City, MO USA; 20 Congenital Heart Center, University of Florida, Gainesville, FL USA; 21 Wolfson Children’s Hospital, Jacksonville, FL USA; 22 Medical College of Wisconsin, Children’s Wisconsin, Milwaukee, WI USA; 23 Department of Pediatrics, Penn State Health, Hershey, PA USA; 24 University of Tennessee Health Science Center, Memphis, TN USA; 25 Case Western Reserve University, University Hospitals Rainbow Babies & Children’s Hospital, Cleveland, OH USA

**Keywords:** Pediatric cardiology, Multidisciplinary conference, Cardiac surgery, Congenital heart disease

## Abstract

Pediatric cardiothoracic surgeries are high-stakes, complex procedures, typically undergoing prior review at multidisciplinary conferences. This study evaluates practice patterns of conferences throughout the United States (US). Surveys were distributed to fellowship program directors or division directors in 124 US pediatric cardiology centers seeking information on conference logistics, fellow roles, quality improvement (QI), and satisfaction. All 47 responding centers (response rate 38%) conduct presurgical conferences, mostly on a weekly basis (92%) lasting 60–120 min (79%). The conferences are solely virtual (19%) or hybrid (81%). High-volume centers (> 300 surgical cases/year) are more likely to hold multiple conferences (13/20 vs 7/27, p < 0.01) and less likely to designate a moderator (11/20 vs 22/26, p = 0.027). Categorical pediatric cardiology fellows at 33 centers present clinical data (97%), echocardiograms (85%), catheterizations (82%), and cross-sectional imaging (39%), typically beginning in their first year. Most centers report that minor (98%) or major changes (51%) are made to patient management at least “sometimes.” Responders rate conferences as very important (median 10/10 on a 10-point Likert scale, IQR 9–10), but satisfaction is more modest (median 7/10, IQR 7–9). Only 17% of centers have a formal QI process. Comments from 42 centers reveal positive themes of collaboration (68%) but also concerns about lengthy (30%) or inefficient (36%) discussion. Conclusions: This survey highlights common practices for pediatric cardiothoracic presurgical conferences. Conferences are collaborative and seen as highly impactful. However, satisfaction varies, and QI efforts are infrequent. These findings highlight opportunities for process improvement and standardization.

## Introduction

Pediatric cardiothoracic surgeries are high-stakes and often complex procedures, typically undergoing prior review at multidisciplinary conferences. These conferences, also referred to as multidisciplinary meetings or surgery boards, are sessions where upcoming surgical cases are reviewed and discussed by a multidisciplinary team, including cardiovascular surgeons, cardiologists, intensivists, anesthesiologists, and multiple other specialists. Such meetings allow for conversation and collaboration between various professionals with the goal of optimizing the perioperative patient care plan. While these conferences have become an integral part of patient care at multiple pediatric cardiovascular surgical centers, they have not, to our knowledge, been formally studied. This study is the first known evaluation of the structure and practice patterns of pediatric cardiothoracic presurgical conferences held at centers across the US.

## Methods 

A thorough literature review was conducted to identify articles addressing the role of multidisciplinary meetings in medical decision making, which included a recent report from the British Society that proposed guidelines [1]. Based on this review, we developed and pilot tested a REDCap survey covering conference logistics, fellow roles, quality improvement (QI) processes, and perceived satisfaction of participants. The survey included 39 multiple choice questions (with up to 21 follow-up questions) as well as five open-ended questions. This survey was exempt from IRB review.

The survey was distributed via email to cardiologists at 124 pediatric cardiology centers in the United States. For centers with a fellowship program, the fellowship director received the survey, and for those centers without fellows, the division director received the survey. This method of distribution allowed for one survey to be completed per center.

Responses were summarized using counts and percentages for multiple choice questions. Comparisons between high- and low-volume centers (defined as greater than or less than 300 cases per year) were performed using Chi Squared tests, with significant p values set a priori at < 0.05. Open-ended questions were individually reviewed, systematically coded by keywords, and finally grouped into common themes as per qualitative analysis guidelines [2].

## Results

### Centers

The survey achieved a 38% response rate from 47 unique centers (Appendix 1). Respondents included 17 pediatric cardiology division directors and 30 pediatric cardiology fellowship directors. Of the responding centers, 20 have high surgical case volumes, 26 have low surgical case volumes, and 1 did not specify case volume.

### Logistics

All respondents’ centers hold a presurgical conference. Most conferences are weekly (92%), in the morning (73%), and 60 – 120 min in duration (79%). No conferences are fully in-person, as 81% (38/47) are hybrid and 19% (9/47) are fully virtual. Some centers (43%, 20/47) hold more than one conference per week, either on a scheduled (15/47, 32%) or ad hoc (4/47, 9%) basis. High-volume centers are more likely to hold more than one weekly conference compared to low-volume centers (13/20 vs 7/27, p < 0.01).

**Fig. 1:**
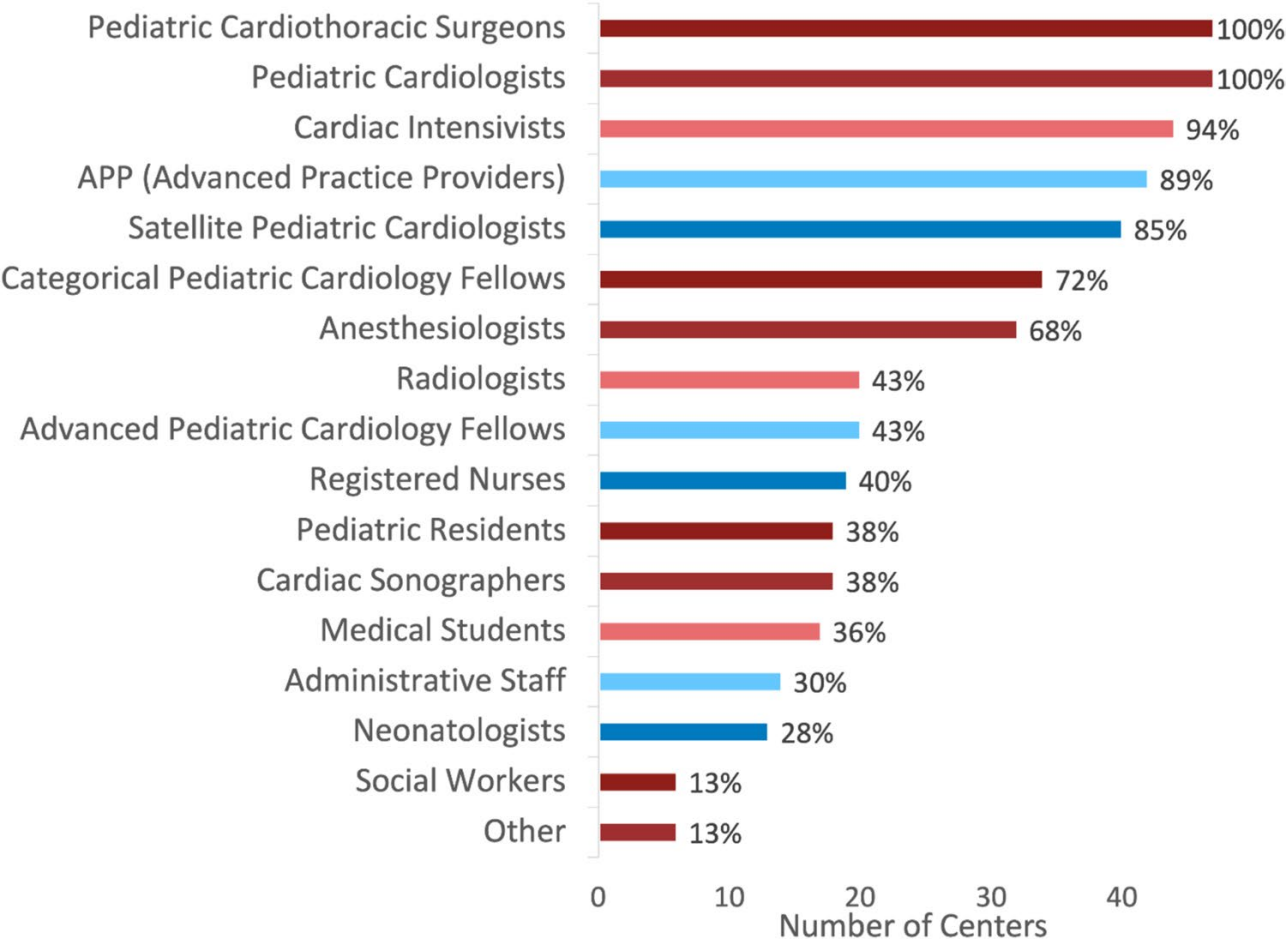
Specialties in attendance at presurgical conferences across responding centers

Conferences are attended by individuals from multiple different specialties and disciplines (Fig. 1). All centers have pediatric cardiothoracic surgeons and pediatric cardiologists in attendance. Most centers also have cardiac intensivists (44/47, 94%), advanced practice providers (42/47, 89%), fellows (34/47, 72%), and anesthesiologists (32/47, 68%) present. A smaller percentage of centers have radiologists (20/47, 43%), registered nurses (19/47, 40%), cardiac sonographers (18/47, 38%), pediatric residents (18/47, 38%), medical students (17/47, 36%), administrative staff (14/47, 30%), and neonatologists (13/47, 28%) in attendance. A minority of centers have social workers (6/47, 13%) and others in attendance, including perfusionists, operating room staff, and catheterization laboratory staff (6/47, 13%).

### Process

**Fig. 2:**
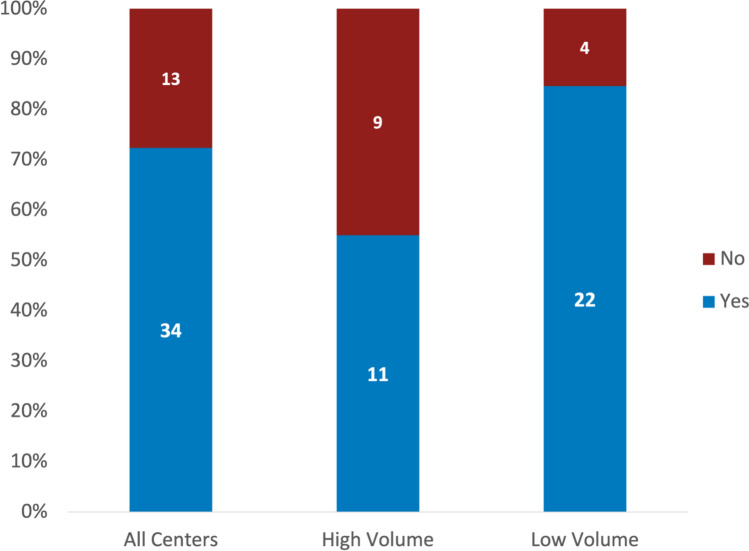
Presence of a designated conference moderator

Moderators were present in 72% (34/47) centers, being more common in low-volume centers (22/26 vs. 11/20, p = 0.027) (Fig. 2). Reported moderator roles included keeping time, guiding discussion, managing virtual chat, and documenting.

A dedicated support person responsible for the administrative duties of the conference is present in 83% (39/47) of centers, with no significant difference between high-volume and low-volume centers.

**Fig. 3:**
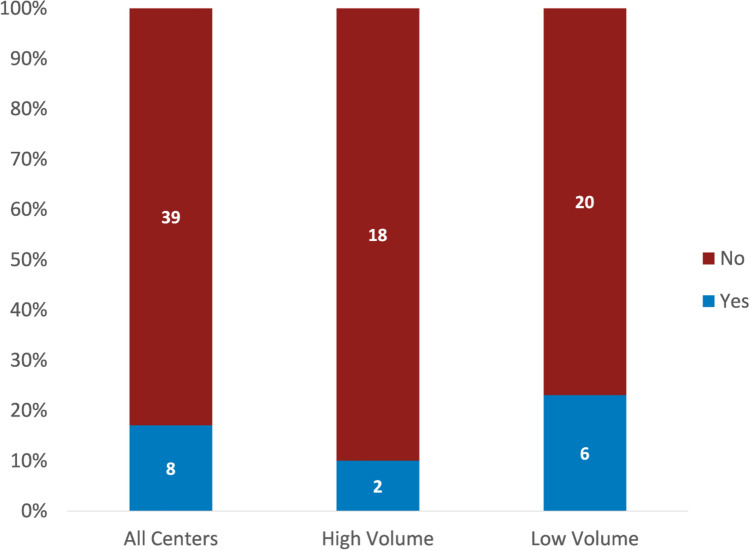
Presence of formal QI process for presurgical conference

A formal QI process for conference is present in only 17% (8/47) of responding centers (Fig. 3). There was no significant difference in the presence of a QI process between low- and high-volume centers. The eight centers with QI efforts reported disparate processes: three centers report procedures comparing surgical or clinical outcomes to conference discussion, while one center reported the QI process remains in development. For the remaining centers, additional QI processes include reviewing imaging data for deficiencies, creating a committee to review conference logistics, distributing an annual survey (though this practice was stopped as no changes had been enacted from survey results).

Seventy-four percent (35/47) of centers record conference discussions in the EMR, compared to 34% (16/47) that maintain a document on file and 8% (4/47) that do not regularly record discussions.

### Outside Center Surgical Referrals

Outside center surgery referral cases are addressed in various manners, with some centers utilizing multiple methods. Most centers directly discuss these cases at conference (27/47, 57%), while a portion of centers have a cardiologist review the cases before presentation at the conference (16/47, 34%). Other centers have a referral team review the case (8/47, 17%), and a few discuss these cases at a separate conference (2/47, 4%).

### Pediatric Cardiology Fellows

**Fig. 4:**
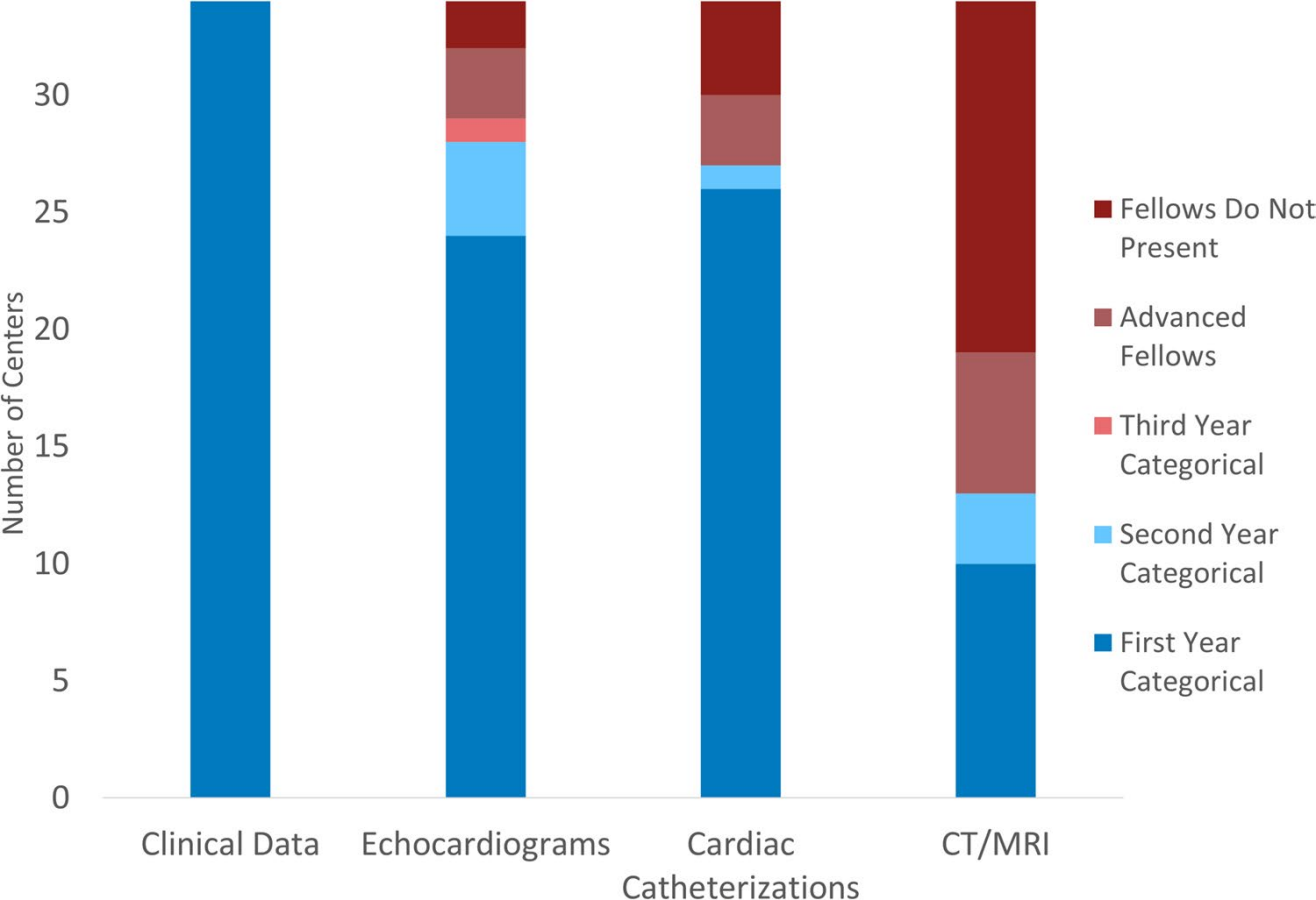
Earliest fellow training level for presenting conference data

Thirty-four of the 47 responding centers (72%) have a categorical pediatric cardiology fellowship training program, and 18 of these centers (18/34, 53%) also train advanced fellows. One center has an advanced fellow but no categorical fellows. Every center with a high surgical case volume had fellows, while only half of low surgical case volume centers had fellows (20/20 vs 13/26, p = 0.0002). Figure 4 demonstrates the training level at which fellows begin to present different types of data during conference. Most centers have categorical fellows begin presenting in their first year: clinical data (34/34, 100%), cardiac catheterizations (26/34, 76%), and echocardiograms (24/34, 71%). A smaller group has second or third years begin presenting imaging and hemodynamic data. Interestingly, many (17/34, 50%) do not have categorical or advanced pediatric cardiology fellows present CT/MRI studies. Centers with and without advanced fellows had similar levels of categorical fellow participation. In centers where there are no fellows or where fellows do not present the data, pediatric cardiologists present most of the data, often with CT/MRI presentations from radiologists.

Centers with fellowship programs were asked to identify the primary goal of conference. Surgical planning was identified as the primary goal in 29% of centers (10/34), while 71% (24/34) of centers identified the primary goal being both education and surgical planning.

### Conference Perception

**Fig. 5:**
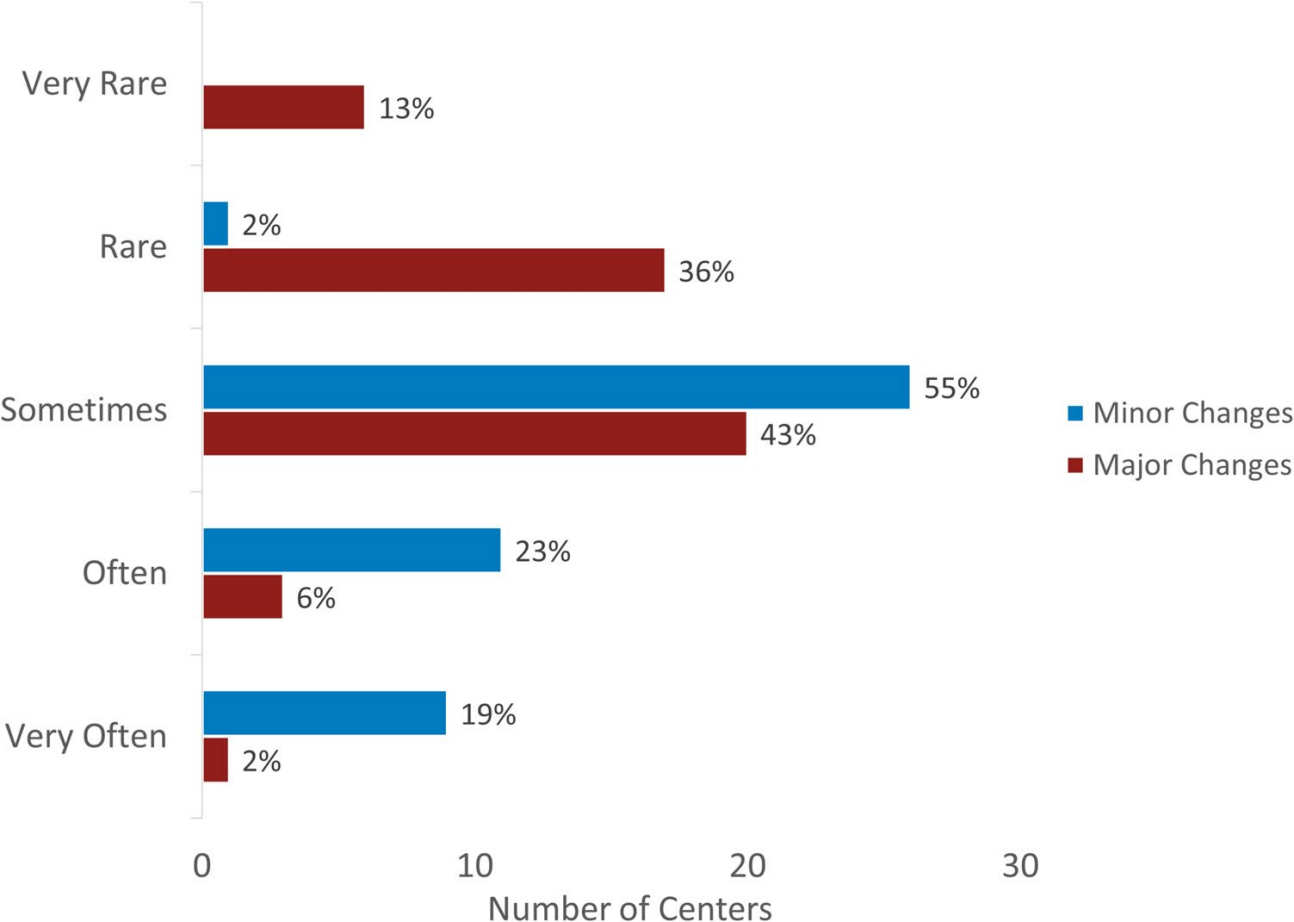
Perceived frequency of *minor* and *major* changes to patient care resulting from conference discussions

In order to evaluate the perceived impact on patient care, respondents were asked to rate the frequency at which conference discussion led to changes in patients’ surgical plans. The majority of centers reported making minor changes to surgical plans *sometimes* (26/47, 55%), *often* (11/47, 23%) or *very often* (9/47, 19%) (Fig. 5). For major changes, there was a wider range of perceived frequency, with the majority feeling that major changes occur *sometimes* (20/47, 43%) or *rarely* (17/47, 36%).

**Fig. 6:**
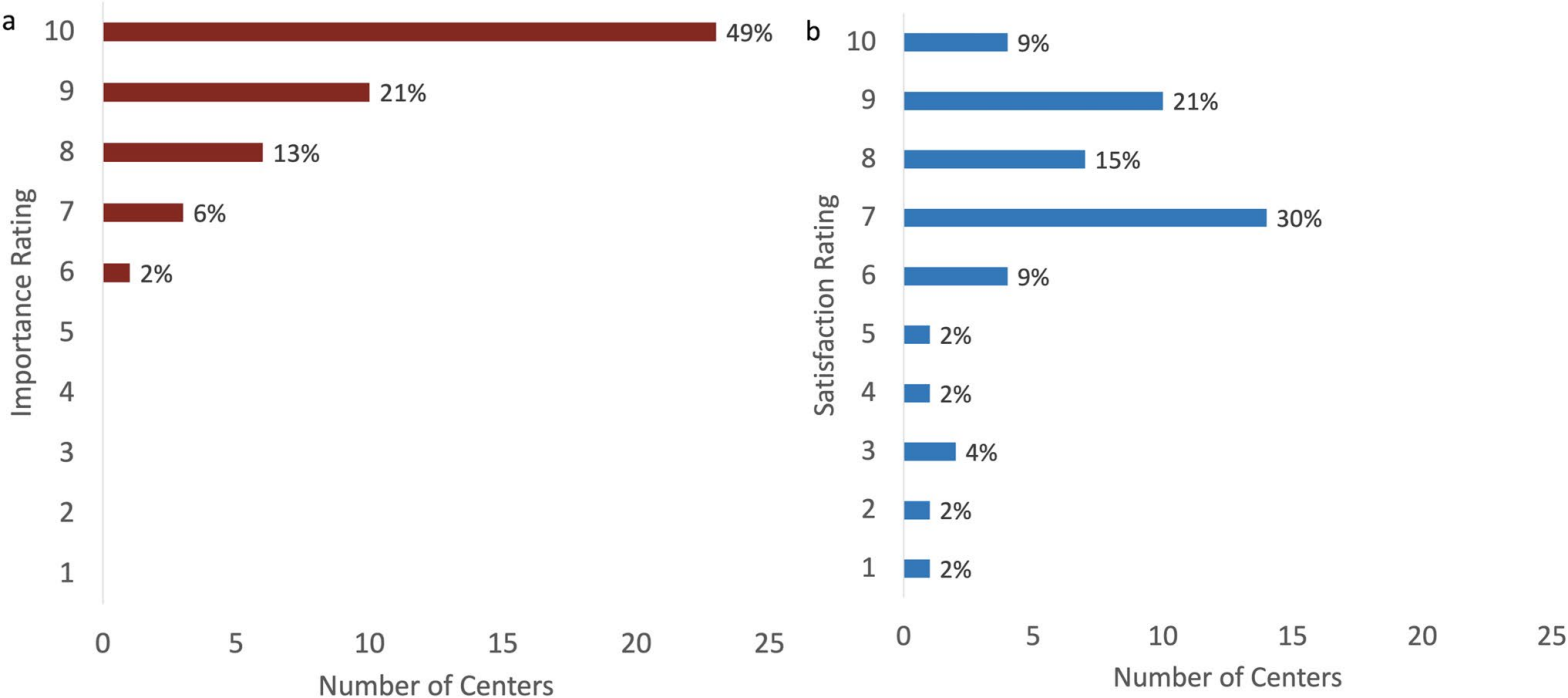
Importance **a** and satisfaction **b** of conference on a 10-point Likert scale with 1 representing the lowest rating and 10 representing the highest rating

Respondents were asked to rate the importance of conference and their degree of satisfaction. Every respondent felt that conference was important, with a median rating of 10 (IQR 9, 10) (Fig. 6). However, the median rating for satisfaction was 7 out of 10 (IQR 7, 9), with a wider range of responses.

**Fig. 7:**
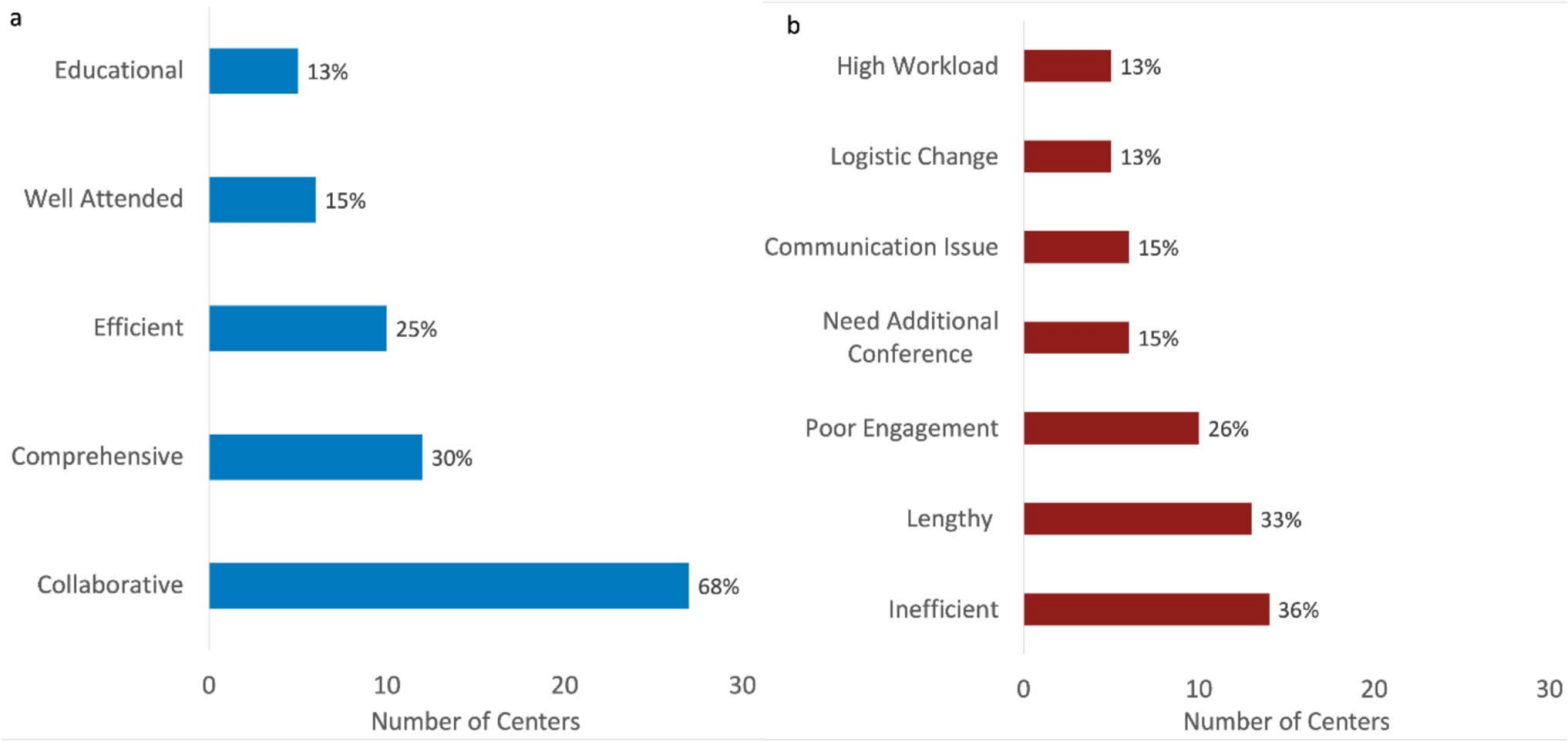
Common themes extrapolated from comments left by respondents regarding aspects of conference that they were pleased with **a** and aspects they would like to change **b**

Several themes emerged from the 40 responses received with open-ended descriptions of aspects of conference respondents were pleased with (Fig. 7a). Most commented on a multidisciplinary, collaborative environment (27/40, 68%), with respondents noting a "healthy group think approach to complex cases" and a “collegial and productive atmosphere.” Multiple respondents (12/40, 30%) felt the comprehensive nature of patient review was a positive aspect, with review that was “thorough” and in a setting where “all pertinent information is available for every patient.” Another positive theme included conference being efficient (10/40, 25%) with “a consistent pattern of presentation” that is “organized” and in a “standard format.”

Additionally, respondents were asked to describe aspects of conference they would like to change and several themes emerged from the 39 responses received (Fig. 7b). It was frequently noted that conferences were lengthy (13/39, 30%), with several mentioning providers needing to leave early for clinical duties before all cases were discussed. Conference felt inefficient (14/39, 36%) at some centers with specific comments mentioning circular discussions, excessive details, repeated presentations, or lack of effective moderation. Some respondents noted poor engagement during conference (10/39, 26%), both due to lack of attendance and lack of participation. Some of these comments stated a preference for in-person meetings to increase engagement and specified that virtual meetings resulted in difficult, disconnected conversations. Complex patients, second opinion cases, and outpatients were mentioned as populations that could benefit from a separate review process. Some commented on a high workload (5/39, 13%) including lack of recognition for or equal distribution of faculty efforts, excessive burden on categorical fellows, and not enough ownership from primary cardiologists. Logistical issues were indicated by some (5/39, 13%) with specific comments regarding mode of conference, time of conference, and timing of patient discussion related to surgery date. Additionally, there were criticisms of communication within the conference (6/39, 15%), including tangential conversations, interruptions or tension-generating outbursts, and use of vague language rather than concrete, data-driven arguments to support decision making.

## Discussion

This is the first known study to systematically examine the structure and practices of multidisciplinary pediatric cardiothoracic presurgical conferences across U.S. centers. Four principal findings emerged from our national survey. First, these conferences are universally utilized, and nearly all respondents view them as critically important to patient care, with a median importance rating of 10 out of 10. Second, there is some variability in both the form and function of these conferences, including differences in frequency, duration, moderation, documentation, and quality improvement efforts. Third, pediatric cardiology fellows are almost universally involved in these conferences at training centers, often taking on substantive roles in data presentation from the first year of fellowship, underscoring their educational value. Fourth, while participants emphasized the collaborative nature and clinical relevance of these conferences, they also highlighted significant barriers to their success, including inefficiency, limited engagement, unclear communication, and inadequate recognition of the time and effort required to participate effectively. These findings provide a foundation for future efforts to optimize presurgical conference structure, efficiency, and impact.

A team-based approach to patient care, with multi- specialty collaboration for shared clinical decision making, is becoming the paradigm across medical specialties, including oncology [3], anesthesia [4], and surgery [5]. Within adult cardiology, heart team clinical decision making has advanced to a Class IC Recommendation in US and European heart guidelines for complex coronary revascularization [6, 7] and valve disease [8, 9]. The British Cardiovascular Societies have gone as far as issuing recommendations for the structure and function of these meetings, including both recommendations that are disease specific as well as general, such as appointing a meeting chair and coordinator to maximize efficiency, opting for a virtual or hybrid platform to increase accessibility, and involving attendees from all fields of patient care [1]. There is evidence that these multidisciplinary meetings for coronary artery intervention are reproducible, feasible, and result in good outcomes aligned with society guidelines [10–13].

There has been far less inquiry thus far in the role of multidisciplinary conferences within pediatrics and congenital heart disease. While multidisciplinary conferences have long been a mainstay of pediatric cardiothoracic surgical programs, no investigation of their format or efficacy has been performed to our knowledge. Given the paucity of existing information regarding this conference, we devised a survey study to better understand practice patterns across the US. Such survey studies have been performed in other medical fields to better understand their multidisciplinary meetings [14, 15]. These survey studies provide a basic understanding of current practices of pediatric cardiothoracic presurgical conferences and serve as a necessary first step toward their improvement or the eventual creation of ‘best practice’ recommendations. Key components of a successful conference could be shared, however, with the caveat of allowing individual innovation that embraces a particular center’s strengths and culture.

Our study demonstrated that all responding pediatric cardiothoracic surgery centers hold multidisciplinary conference, likely a result of the longstanding close relationship between numerous specialists caring for this population as well as the unique and complex nature of many congenital heart disease patients. We found that many centers conduct these conferences in a logistically similar manner with similar time frames, virtual options, and EMR documentation. There are typically a wide range of specialists in attendance, with nearly all centers involving cardiothoracic surgeons, pediatric cardiologists, pediatric critical care physicians, and advanced practice providers, but there was variability in the participation of anesthesiologists, radiologists, and neonatologists. Pediatric cardiology fellows are typically highly involved and responsible for presentation of clinical, echocardiographic, and catheterization data, usually starting in their first year of categorical fellowship, but they less frequently present cardiac cross-sectional imaging. While 71% of centers with pediatric cardiology fellowship programs indicated that education is a primary goal of conference, only 13% of positive comments were related to education. Furthermore, respondents reported common themes of conferences being “lengthy” and “inefficient,” suggesting that there may be a tension to incorporate education into the already prolonged conference discussions. Identifying best practices to balance education and with fellow workload and conference time constraints is of importance.

Conference discussion has a strong perceived impact on patient care. Nearly every center reported that changes to the care plan are made regularly at multidisciplinary conferences, with half reporting *major* changes to patient care occur “sometimes,” “often,” or “very often.” In comparison, formal studies of adult cardiology multidisciplinary conferences have reported changed patient management in 30–54% of cases [16, 17], and a meta-analysis has shown adult cardiology multidisciplinary conferences may provide benefit to patient outcomes [13]. A large meta-analysis of over 130,000 oncology patients demonstrated that multidisciplinary conferences increased overall survival with a hazard ratio of 0.67 [3]. Dedicated, prospective studies would be needed to truly understand the frequency of changes to care due to pediatric cardiothoracic presurgical conference discussion, which may not be possible given that conferences have already become the standard of care.

In addition to a perceived impact on patient care, respondents universally felt that these conferences were important with a median rating of 10 on a 10-point Likert scale (IQR 9, 10). However, despite every participant rating conference as important (defined as a score above 5), the satisfaction ratings were highly variable, with a median rating of 7 out of 10 (IQR 7,9) and every possible score selected (1–10). QI efforts were not frequently implemented and often lacked formal, structured QI methodology when present. This suggests that there may be opportunities for QI and sharing best practices from centers with high conference satisfaction.

We sought to gather qualitative hypothesis-generating themes on respondents’ perceptions of surgery conference through open-ended questions. Comments had recurrent themes of strong collaborative efforts and supportive environments for discussion. However, another major theme was inefficiency, with multiple mentions of circular discussions and inadequate time. It was noted that physician’s time spent on these conferences is typically not compensated, and the work involved was sometimes felt to be not fairly distributed. Addressing these aspects of conference may serve as a starting point to improve satisfaction. Additional efforts for QI may draw from adult cardiology multidisciplinary recommendations [13], including defined roles and responsibilities, robust communication, decision making and evaluation protocols, and patient centric approaches. Furthermore, there is growing awareness that cognitive bias may hinder rational decision making in these conferences [18], and efforts to evaluate and minimize these biases may also be important to improving conference success. A landmark paper from Dublin brings to light inconsistencies in decision-making during pediatric cardiothoracic presurgical conferences, highlighting the complexity of cases, absence of evidence-based data and impact of cognitive bias as contributing factors [19]. They suggest that validated decision-making algorithms may enable consistency in decision making, which is an area of study that warrants further research moving forward.

It is important to note several limitations to this study. As with all surveys of this type, the results have the significant limitation of recall, response, and confirmation bias. They are also limited by confirmation bias, although effort was made to use neutral and objective language in the survey to help limit the effects of this bias. It also reflected the viewpoint of pediatric cardiologists and not that of other providers that contribute to these multidisciplinary conferences. However, our methods ensured that there was only one response per center completed by a person in a leadership position. It is also worth mentioning that respondents were left to define minor and major changes to the surgical plans, and the details of these changes were not elicited nor specified as operative or perioperative. Finally, the impact of conferences on patient care was not directly evaluated. Finally, the response rate was only 38%, but fortunately still encompassed programs of both high and low volume.

This survey brings transparency to the practice patterns of cardiothoracic presurgical conferences across US pediatric cardiology surgical centers. However, this is a first step toward critical examination of these important programs. More granular, site-specific QI studies can be helpful to identify specific barriers to more efficient, satisfactory, and efficacious presurgical conferences.

## Conclusion

Pediatric cardiothoracic presurgical conferences are nearly universal at pediatric surgical centers and have similar logistical patterns. These conferences are universally seen as important but have variable satisfaction ratings. Common themes suggest room for improvement in conference efficiency, attendance, and length. Better understanding of conference practice patterns across centers may help with critical evaluation and improvement of these complex and impactful conferences.

## Data Availability

No datasets were generated or analysed during the current study.

## Appendix

1. Responding Centers:
2. Alabama, Children’s of Alabama, University of Alabama Birmingham, Birmingham
3. Arizona, Phoenix Children’s, Phoenix
4. Arkansas, Arkansas Children’s Hospital, University of Arkansas for Medical Sciences, Little Rock
5. California, Children’s Hospital of Orange County, Orange County
6. California, MemorialCare Miller Children’s and Women’s Hospital, Long Beach
7. California, UC Davis Children’s Hospital, Sacramento
8. California, University of California San Diego, San Diego
9. California, University of California San Francisco, San Francisco
10. California, Valley Children’s Heathcare, Madera
11. Colorado, Rocky Mountain Hospital for Children, Denver
12. Colorado, University of Colorado Denver, Denver
13. Delaware, Nemours Cardiac Center, AIDHC, Wilmington
14. Florida, Congenital Heart Center University of Florida, Gainesville
15. Florida, Johns Hopkins All Children's Hospital, St. Petersburg
16. Florida, Wolfson Children’s Terry Heart Institute, Jacksonville
17. Georgia, Emory University, Atlanta
18. Illinois, Ann & Robert H. Lurie Children’s Hospital of Chicago, Chicago
19. Illinois, Rush University Medical Center, Chicago
20. Illinois, University of Illinois College of Medicine Peoria, Peoria
21. Indiana, Riley Children’s Hospital at Indiana University Health, Indianapolis
22. Iowa, University of Iowa Carver College of Medicine Iowa City, Iowa City
23. Kentucky, University of Kentucky College of Medicine, Bowling Green
24. Kentucky, University of Louisville, Louisville
25. Louisiana, Louisiana State University, Baton Rouge
26. Maine, Maine Medical Center, Portland
27. Michigan, University of Michigan C.S. Mott Children’s Hospital, Ann Arbor
28. Minnesota, Mayo Clinic, Rochester
29. Missouri, Children’s Mercy Kansas City, Kansas City
30. Missouri, Saint Louis University School of Medicine, SSM Health Cardinal Glennon Children’s Hospital, Saint Louis
31. Nebraska, University of Nebraska Medical Center, Children’s Nebraska, Omaha
32. New York, New York University Grossman School of Medicine, New York
33. New York, Pediatric Cardiology Associates of CNY, Upstate Medical University, Syracuse
34. New York, University of Rochester Medical Center, Rochester
35. North Carolina, Duke University, Durham
36. North Carolina, University of North Carolina at Chapel Hill, Chapel Hill
37. Ohio, Cleveland Clinic Children’s Hospital, Cleveland
38. Ohio, UH Rainbow Babies & Children’s Hospital, Case Western Reserve University, Cleveland
39. Oregon, Oregon Health Sciences University, Portland
40. Pennsylvania, Penn State Health Children’s Hospital, Hershey
41. Pennsylvania, The Children’s Hospital of Philadelphia, Philadelphia
42. Pennsylvania, UPMC Children’s Hospital of Pittsburgh, Pittsburgh
43. South Carolina, Medical University of South Carolina, Charleston
44. Tennessee, University of Tennessee Health Science Center, Memphis
45. Texas, Driscoll Children’s Hospital, Corpus Christi
46. Texas, Methodist Children’s Hospital San Antonio, San Antonio
47. Texas, University of Texas Health at Houston, Houston
48. Wisconsin, Medical College of Wisconsin, Children’s Wisconsin, Milwaukee
